# Predicting lung adenocarcinoma prognosis with a novel risk scoring based on platelet-related gene expression

**DOI:** 10.18632/aging.202682

**Published:** 2021-02-22

**Authors:** Chengmao Zhou, Yongsheng Wang, Ying Wang, Lei Lei, Mu-Huo Ji, Guoren Zhou, Hongping Xia, Jian-Jun Yang

**Affiliations:** 1 Department of Anesthesiology, Pain and Perioperative Medicine, The First Affiliated Hospital of Zhengzhou University, Zhengzhou 450000, China; 2 Department of Pathology, School of Basic Medical Sciences & Key Laboratory of Antibody Technique of National Health Commission & Jiangsu Antibody Drug Engineering Research Center, Nanjing Medical University, Nanjing 211166, China; 3 Department of Respiratory Medicine, Affiliated Drum Tower Hospital of Nanjing University Medical School, Nanjing 210008, China; 4 Jiangsu Cancer Hospital, The Affiliated Cancer Hospital of Nanjing Medical University, Jiangsu Institute of Cancer Research, Nanjing 210009, China; 5 School of Medicine, Southeast University, Nanjing 210009, China; 6 Sir Run Run Hospital, Nanjing Medical University, Nanjing 211166, China

**Keywords:** lung adenocarcinoma, bioinformatics analysis, TCGA, platelet, nomogram

## Abstract

Lung adenocarcinoma is the most common subtype of non-small cell lung cancer, and platelet receptor-related genes are related to its occurrence and progression. A new prognostic indicator based on platelet receptor-related genes was developed with multivariate COX analysis. Prognostic markers based on platelet-related risk score perform moderately in prognosis prediction. The functional annotation of this risk model in high-risk patients shows that the pathways related to cell cycle, glycolysis and platelet-derived related factors are rich. It is worth noting that somatic mutation analysis shows that TTN and MUC16 have higher mutation burdens in high-risk patients. Moreover, the differential genes of high- and low-risk groups are regulated by copy number variation and miRNA. And we provide a free online nomogram web tool based on clinical factors and the risk score (https://wsxzaq.shinyapps.io/wsxzaq_nomogram/). The score has been verified among three independent external cohorts (GSE13213, GSE68465 and GSE72094), and is still an independent risk factor for lung adenocarcinoma. In addition, among the other 6 cancers, the OS prognosis of high and low-risk groups of PRS is different (P < 0.05). Our research results have screened multiple platelet differential genes with clinical significance and constructed a meaningful prognostic risk score (PRS).

## INTRODUCTION

Global data show that approximately 1.5 million patients die of lung cancer each year, with a mortality rate over 25% [1]. Lung cancer is divided into small cell lung cancer and non-small cell lung cancer (NSCLC), with the latter accounting for 85% of total cases [2]. Lung adenocarcinoma (LUAD) accounts for 30–35% of all lung cancers, and about 500,000 people die of LUAD each year [3]. Although targeted drugs such as ALK and EGFR-TKI have survival benefits for LUAD patients with sensitive gene mutations, the survival time for advanced LUAD patients without sensitive gene mutations remains short. Thus, more treatment targets need to be identified to provide advanced LUAD patients with more effective treatment options and to prolong survival time.

In recent years, the incidence of adenocarcinoma has proliferated, and adenocarcinoma has replaced squamous cell carcinoma as the most prevalent type of NSCLC [4]. LUAD progresses rapidly with a high degree of malignancy. Patients who show clinical symptoms and signs are often at an advanced stage, causing an onerous burden on both themselves and society. Studies [5, 6] have shown that platelets play a non-negligible role in tumor metastasis, and there is a correlation between platelet count and cancer prognosis. Platelets are known to be involved in pathophysiological processes such as inflammation, vascular hemostasis, thrombosis and atherosclerosis [7]. There is also considerable evidence that platelets aggregate and activate malignant tumors [8]. They are also associated with distant tumor metastasis. Antiplatelet drugs (such as heparin) inhibit tumor metastasis, suggesting that inhibiting platelet activation may become a target for tumor treatment.

Potential mechanisms regarding platelet-associated receptors and LUAD prognosis may involve epigenetic changes and genetic mutations. Therefore, it is of clinical significance to build molecular models of platelet-associated receptor genes that predict LUAD prognosis and have the potential to guide personalized clinical treatment strategies. In this study, we developed a specific, accurate, and reliable prognostic risk scoring system based on platelet-associated receptor genes for LUAD. We also performed bioinformatics analysis to explore potential regulatory mechanisms.

## RESULTS

### Identifying differentially expressed genes (TCGA)

**Figure 1:**
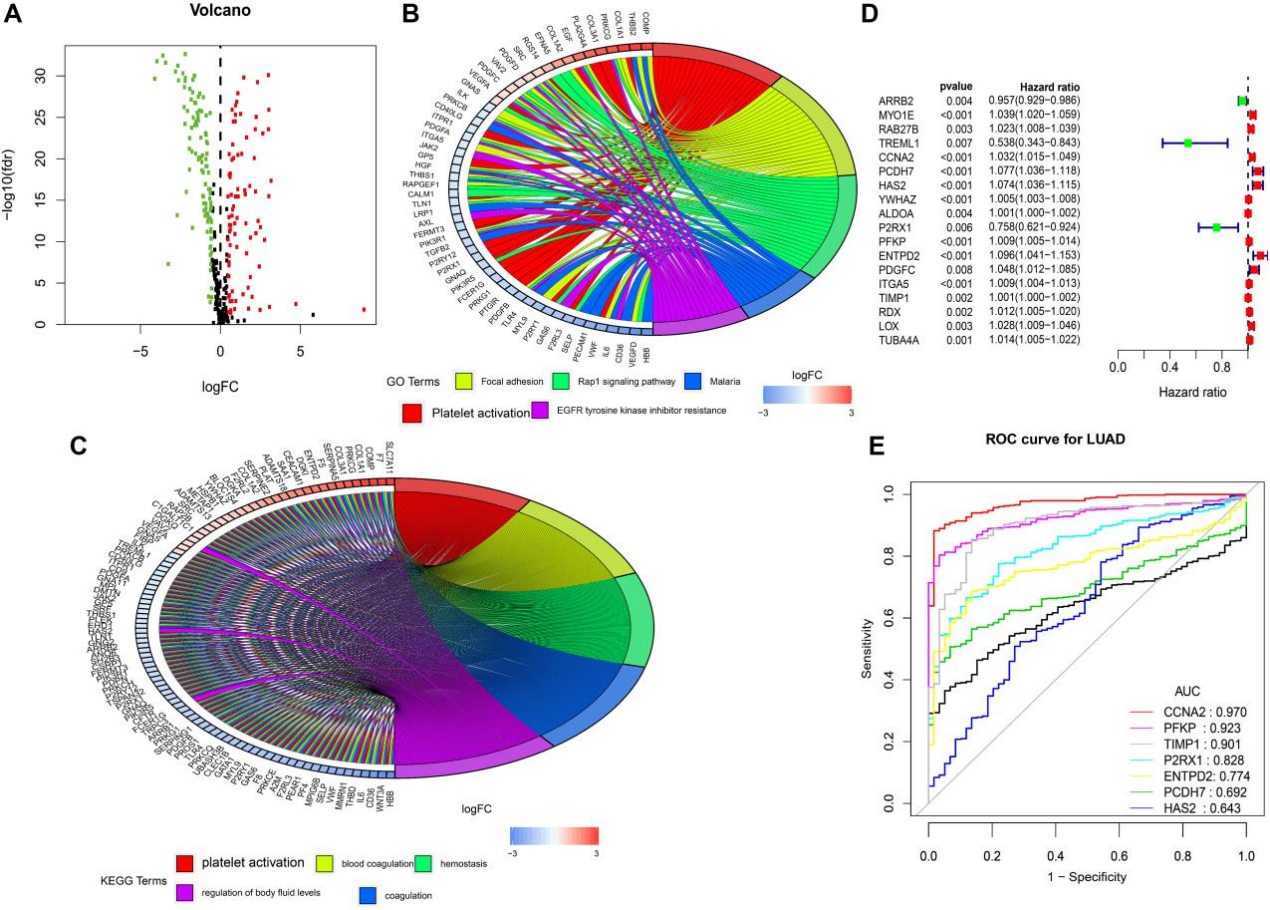
**Differentially expressed platelet receptor-related genes, Gene functional enrichment and identification of hub platelet receptor-related genes.** (**A**) Volcano plot of differentially expressed platelet receptor-related genes (DEPG) in the TCGA. (**B**) GO enrichment analysis of DEPG. (**C**) KEGG enrichment analysis of DEPG. (**D**) Single-factor analysis for DEPG. (**E**) Diagnosing lung adenocarcinoma patients.

The EdgeR algorithm can identify 188 differentially expressed genes, 83 up-regulated genes and 105 down-regulated genes (Figure 1A). As expected, gene function enrichment analysis showed that the platelet pathway was the most common. Platelet activation is one of the most common biological terms in biological processes (Figure 1B). And platelet activation is most often enriched by differentially expressed genes. (Figure 1C).

### Identifying differential genes related to survival

After a single factor analysis, we found that 18 differences are related to lung adenocarcinoma patients’ overall survival rates, and these results are statistically significant (Figure 1D). The forest map shows that most of the identified central genes in the lung adenocarcinoma samples are up-regulated. The forest map of hazard ratios shows that most of these genes are risk factors. Nine genes were selected by multifactor regression, but because the validation group did not have the expression value of TREML1, we finally included only the other eight genes into the study. And we constructed a prognostic marker and divided lung adenocarcinoma patients into two groups. The calculation formula is as follows: Platelet-related risk score (PRS) = RAB27B *0.017537331+CCNA2*0.020495528+PCDH7 *0.038831123+HAS2*0.062731768+P2RX1*0.173961877+PFKP*0.006157281+ENTPD2*0.065271557+TIMP1*0.000897402

Platelet-related receptor genes based on multivariate Cox may be an important tool for diagnosing lung adenocarcinoma patients according to potential, and the ROC curve of CCNA2 is 0.970. (Figure 1E).

**Figure 2:**
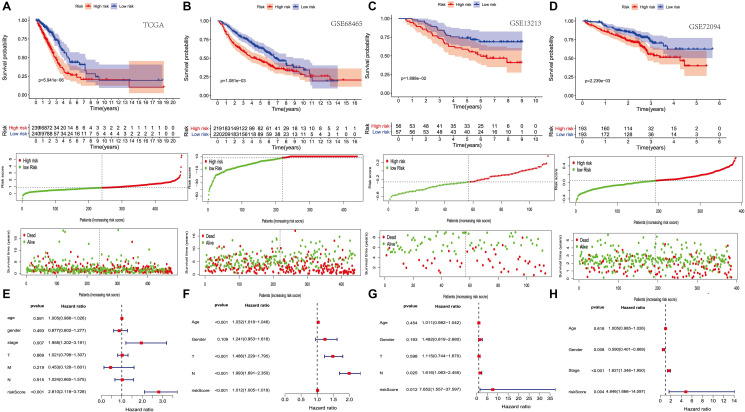
**The prognostic value of PRS (platelet receptor-related risk score).** (**A–D**) Kaplan-Meier curves of overall survival based on the PRS in the TCGA, GSE13213, GSE68465 and GSE72094 cohort (**E–H**) Multivariate Cox regression analysis in the TCGA, GSE13213, GSE68465 and GSE72094 cohort.

Survival analysis showed that the high-risk group’s survival rate was lower than that of the low-risk group, and these results were statistically significant. Figure 2A represents a risk map, including signature-based, individual survival status between groups and the expression levels and distributions of the included modeling genes. It shows a clear difference in survival status between the risk groups, with red dots indicating death and green dots indicating survival. Many deaths occurred in the high-risk group, while most patients in the low-risk group survived through follow-up. In addition, PRS scores are verified in three other independent queues (GSE13213, GSE68465 and GSE72094), and the results are consistent with those of TCGA (Figure 2B–2D). Multivariate Cox regression analysis showed that after adjusting for other parameters like stage and T, the prognostic index can become an independent predictor (Figure 2E). Moreover, multiple Cox regression analysis showed that in GSE13213, GSE68465 and GSE72094 data sets, the prognosis index could also become an independent prediction index (Figure 2F–2H).

**Figure 3:**
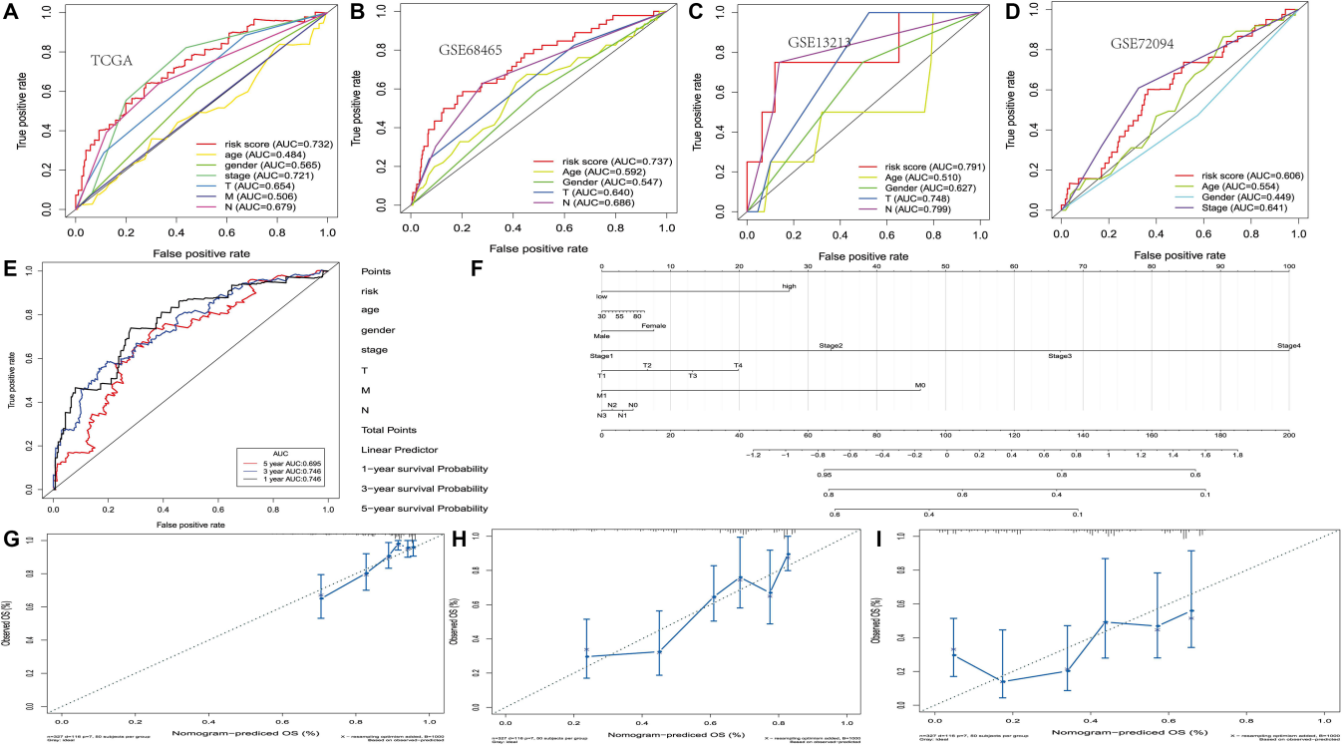
**Development of the nomogram based on PRS and clinical features.** (**A–D**) The receiver operating characteristic (ROC) curve of OS in TCGA, GSE13213, GSE68465 and GSE72094 cohort. (**E**) ROC curve of the nomogram based on PRS in TCGA. (**F**) The nomogram based on PRS in TCGA. (**G–I**) 1, 3 and 5-year calibration curves of nomogram in TCGA.

The ROC curve was 0.732, indicating that there may be a medium potential for PRS based prognosis in survival monitoring. (Figure 3A) There are still survival differences between the high-risk and low-risk groups in the other three datasets (GSE13213, GSE68465 and GSE72094), and the ROC curve values formed by PRS scores are 0.791, 0.737 and 0.606, respectively (Figure 3B–3D). It was also found that for different lung cancer patient subtypes, the platelet-associated gene risk score was a moderately viable indicator for prognosis. (Supplementary Figure 1).

Using the above eight genes and clinical factors for lung cancer, we developed a nomogram to predict the 1-year, 3-year, and 5-year overall survival. The ROC analysis showed sufficient discrimination with an AUC as 0.746, 0.746 and 0.695, indicating that the nomogram has medium prediction performance (Figure 3E and Figure 3F). Besides, the calibration chart showed the best prediction accuracy, and the predicted survival rate was equal to the actual survival rate (Figure 3G–3I). Moreover, we constructed the nomogram by combining PRS with clinical factors, and provided a free nomogram prediction web tool for clinicians (https://wsxzaq.shinyapps.io/wsxzaq_nomogram/) (Supplementary Figure 2).

### Correlation analysis

**Figure 4:**
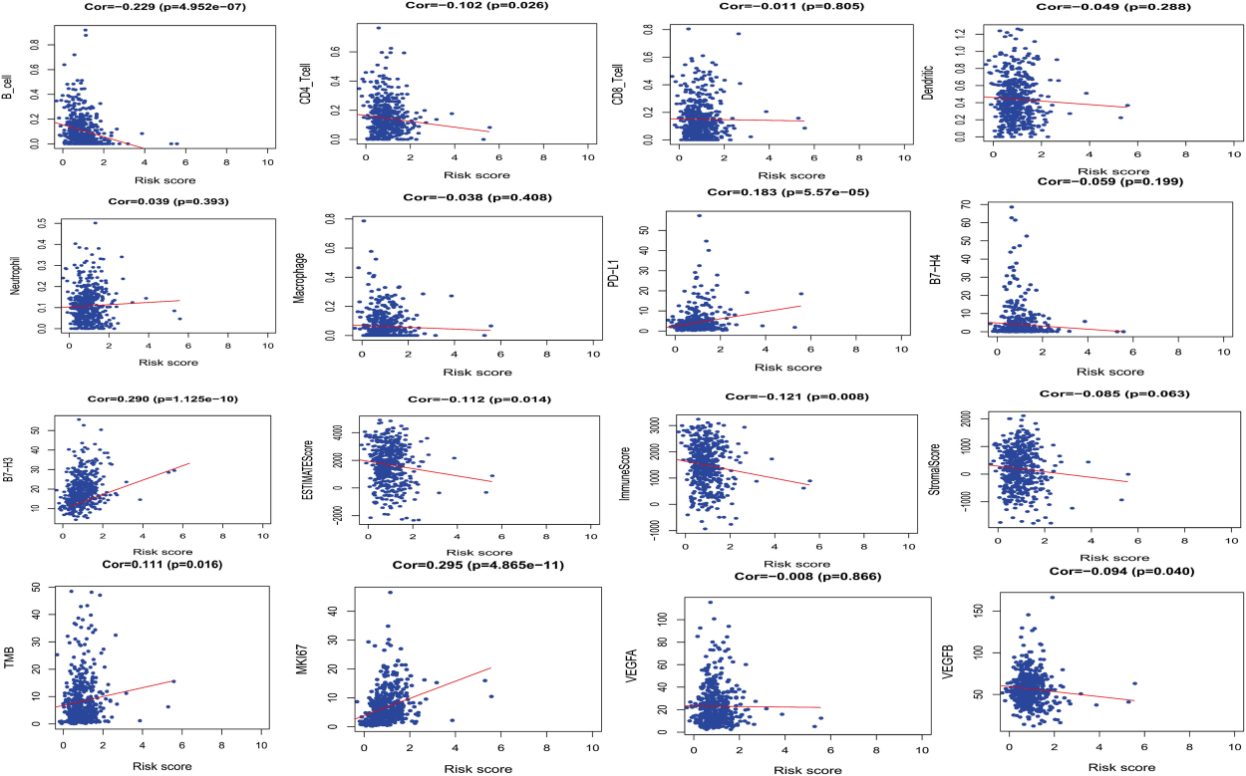
**The relationships between PRS and other prognostic features (Immune infiltration, immune checkpoints and cell proliferation-related genes, etc.).**

Correlation analysis between PRS and immune cell infiltration showed that PRS and B_cell or CD4 T_cell (Cor = –0.229, *P* = 4.952e-07 and Cor = –0.102, *P* = 0.026) were related, and correlation analysis between PRS and immune checkpoints showed that PRS and PD-L1 (Cor = 0.183, *P* = 5.575e-05) were related. PRS was negatively correlated with ESTIMATE score (Cor = –0.112, *P* = 0.014), indicating that PRS was positively correlated with tumor purity of LUAD. PRS was positively correlated with the MKI67 score, which proves that the high-risk group may be closely related to the regulation of cancer cell cycle and cancer cell proliferation (Cor = 0.295, *P* = 4.865e-11). (Figure 4).

### Functional annotation of established eight gene signatures

**Figure 5:**
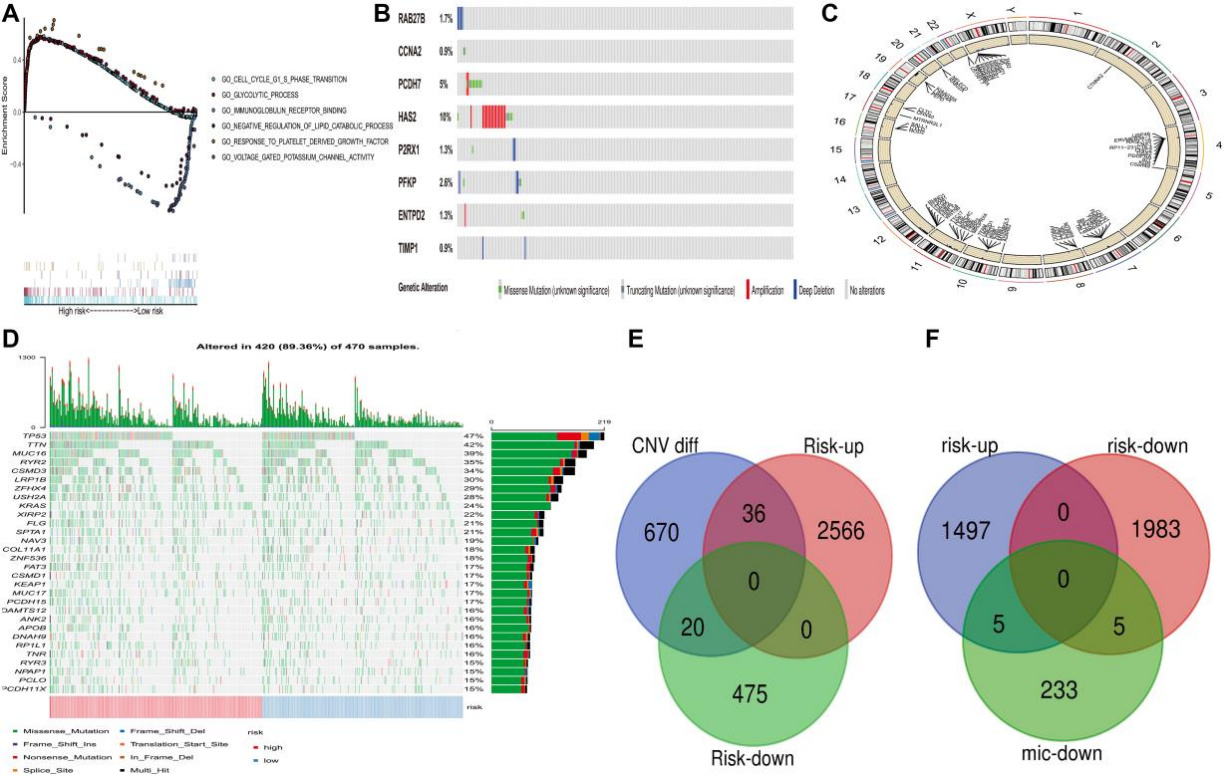
**Genome-wide analysis of PRS risk score. (including mutation, copy number change and microRNA change, etc.).** (**A**) GSEA was performed in TCGA high-risk populations. (**B**) Mutation of 8 Factors Constructed into Risk Score in CBioportal. (**C**) Differential copy number in PRS high and low group. (**D**) Distribution of the First 30 Mutant Genes in High and Low-Risk Groups. (**E**) Overlapping of Up-regulated and Down-regulated Genes for Copy Number Change Genes and Differences between High and Low-Risk Groups (**F**) Downregulated MicRNA target control gene and overlapping gene of up-regulated and down-regulated genes with a high and low-risk difference.

GSEA was performed in TCGA high-risk populations to study key biological and cellular processes related to prognosis. There were clear abundant GO pathways in high-risk patients, including cell cycle, glycolysis and platelet-derived related factors (Figure 5A). Most of these pathways were involved in cell proliferation and migration and may lead to lung cancer metastasis and recurrence. Previous studies have shown that platelet-derived growth factors regulate glycolysis and therefore affect the cell cycle of cancer cells. This may also be a potential mechanism for high-risk PRS, suggesting poor LUAD prognosis.

### Analyzing mutations and CNV in patients with high and low PRS expression

CBioportal database (http://www.cbioportal.org/) shows that among the 8 genes, HAS2 has the highest mutation rate of 10%. (Figure 5B) TTN and MUC16 were detected with high mutation frequencies in tumors expressed in the high-risk group, and they are well-known tumor-associated genes (Figure 5D). This demonstrates that PRS distinguishes mutant genes with poor prognosis. Figure 5C shows the differential copy number in PRS high and low risk, which get copy number in chromosomes 10, 11, 12 and 20, and mitigated in 8, 10, 11 and 12. Among them, the copy numbers of 36 and 20 genes in the high and low-risk groups, respectively, also changed (Figure 5E). The differential expression genes of PRS between high and low expression levels are affected by copy number variation, which indicates that there is no independence between the abnormal copy number expression genes and the differential expression genes of PRS.

### The correlation between miRNA and risk score differential expression

MiRNA is a small non-coding RNA, which can be involved in targeted mRNA cleavage and post-transcriptional regulation. Previous studies have shown that miRNA expression imbalance is a key step in lung adenocarcinoma carcinogenesis. Our work evaluated the relationship between miRNA target-controlled down-regulated genes and risk score differentially expressed genes. Therefore, we used TargetScan to predict the relationship between miRNA and its target genes. A total of 120 up-regulated miRNA and 14 down-regulated miRNA were detected in the high-risk group (the low-risk group served as a reference). We identified 233 pairs of down/miRNA-mRNA interactions by TargetScan (i.e. the miRNA and target control gene scores were greater than or equal to 0.6), of which 10 pairs overlapped with risk score differential expression (Figure 5F). Overlapping genes, including XCT and UCK2, are adverse prognostic factors of lung adenocarcinoma, and the mechanism may be related to regulating the cell cycle of cancer cells. These results indicate that miRNA in the down-regulated group has feedback loops in the carcinogenesis processes of the high-risk and low-risk groups.

### External data support

**Figure 6:**
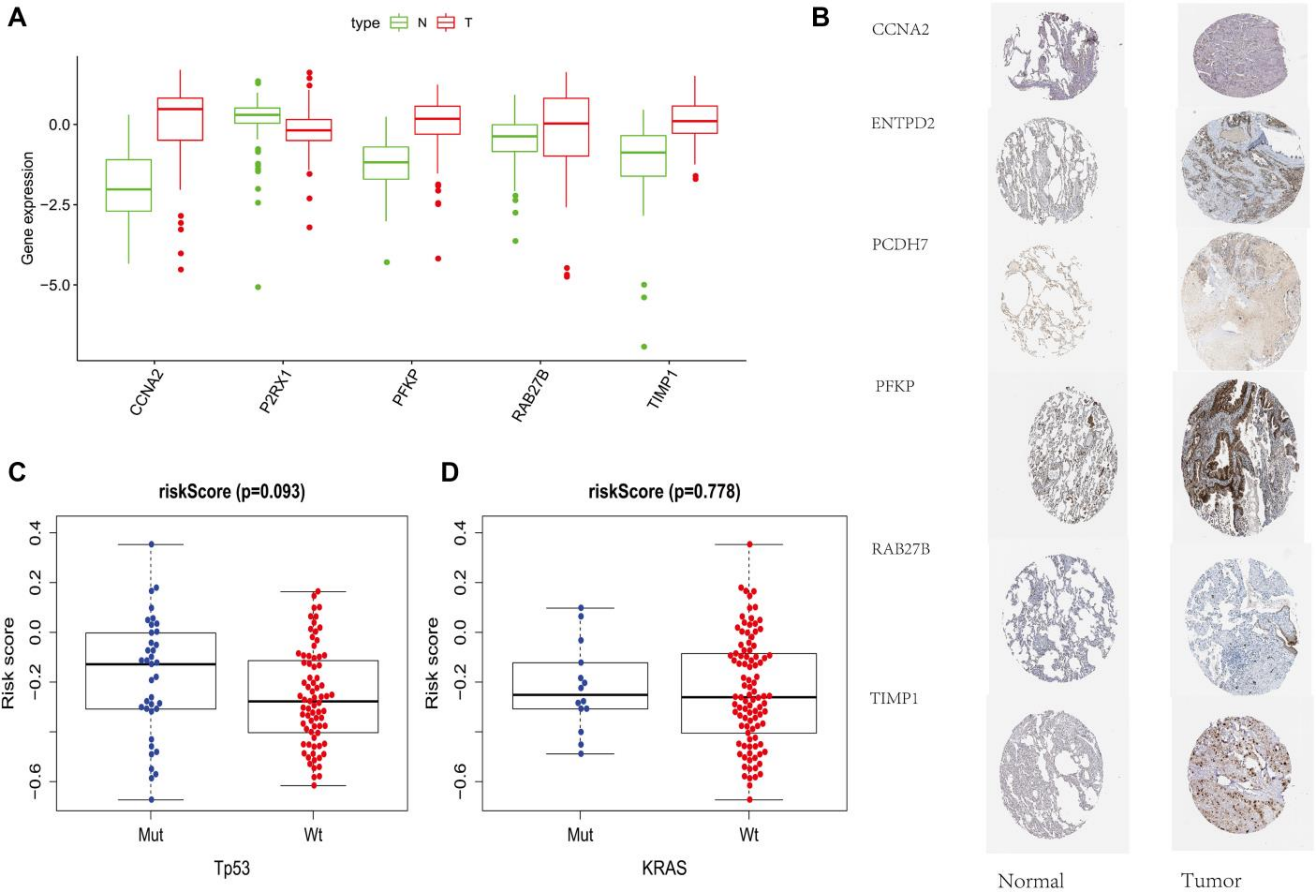
**External data support.** (**A**) Protein Expression of Risk Score Components in TCPA Database. (**B**) Immunohistochemical Results of Risk Score Components in HPA Database. (**C**) The relationship between PRS and TP53 mutations and KRAS mutations on GSE13213.

By analyzing the HPA (http://www.proteinatlas.org) and TCPA (http://tcpaportal.org) protein databases, we found that the protein expression of related genes constructed by PRS was also consistent with the previous results. (Figure 6A and 6B) Finally, we verified the relationship between PRS and TP53 mutations and KRAS mutations on GSE13213 data sets and found that the risk score was not affected by these two gene mutations. (Figure 6C and 6D).

In addition, among the other 6 cancers, the high-risk scoring group of PRS also has a significantly worse prognosis than the low-risk group, and the difference is statistically significant (*P* < 0.05). (Supplementary Figure 3).

## DISCUSSION

This study has identified and verified platelet receptor-related gene risk scoring systems for predicting LUAD’s OS. After a comprehensive analysis, 188 genes were selected as the final specific genes. Finally, eight prognostic gene signatures (PRS: RAB27B, CCNA2, PCDH7, HAS2, P2RX1, PFKP, ENTPD2 and TIMP1) were constructed by COX multivariate regression model and verified in three other independent datasets (GSE13213, GSE68465 and GSE72094). Besides, GSEA enrichment analysis showed that high-risk group scores may affect cell cycle, glycolysis and platelet-derived related factors. Moreover, the potential mechanism of PRS risk score in lung adenocarcinoma prognosis was comprehensively analyzed.

At present, studies on these gene-related cancers have been reported. Studies [9] have shown that Rab27B regulates the aggressive growth and metastasis of ER-positive breast cancer cell lines, and its increased expression is associated with poor prognosis. Similarly, a high expression of Rab27b is associated with poor LUAD prognosis, which may be a potential indicator of LUAD metastasis and poor prognosis [10]. The SNP (rs769236) on the CCNA2 promoter may be associated with an increased risk of colon, liver, and lung cancer [11]. Additionally, HAS2 may be involved in the aggressive phenotypes of primary breast cancer [12]. The simultaneous silencing of HA synthase 2 (HAS2) and HAS3 or CD44 and RHAMM inhibits cell proliferation and survival, as well as the EGFR/AKT/ERK signaling pathway [13]. It has been shown that PFKP is one of the differential lncRNA-miRNA-mRNA networks in NSCLC, and it is related to glycolysis [14]. In hepatocellular carcinoma, hypoxia induces the expression of ENTPD2 on cancer cells; This increases extracellular 5'-AMP, and in turn, promotes MDSC maintenance by preventing its differentiation [15]; moreover, studies have shown that ENTPD2 is of great significance in the diagnosis, monitoring and prognosis of LUAD [16]. Inhibiting TIMP1 expression reduces proliferation and metastasis and increases apoptosis. TIMP1 mRNA expressed in the platelets of colorectal cancer patients can be transported into colorectal cancer cells, thereby promoting tumor growth [17]. The meta-analysis also shows that highly-expressed TIMP-1 is associated with poor prognosis in NSCLC patients [18]. In addition, studies have shown that P2RX1 is one of the prognostic genes of LUAD [19].

Gene enrichment analysis of high-risk patients also shows that the pathways related to cell cycle, platelet-derived growth factor and glycolysis were enriched. Studies have shown that CCNA2 [20], HAS2 [21] and TIMP1 [22] can regulate the cell cycle of cancer cells. Besides, studies [23] have also shown that PFKP can affect the proliferation of cancer cells through the glycolytic pathway. Thus, platelet-related receptor genes in lung adenocarcinoma may affect the proliferation and metastasis of lung adenocarcinoma by regulating cell cycle and glycolysis. And the potential mechanism of distinguishing lung adenocarcinoma by this risk score may be related to copy number variation and negative feedback by miRNA and may be related to regulating the cell cycle.

Many studies have shown that these eight genes play an important role in the biological function of platelets. Rab27b has been a key modulator of the secretion of dense particles in platelets [24]. Studies have shown that there is a close relationship between CCNA2 and plasma release of platelets [25]. Platelet-derived growth factors modulate the activity of HAS2 [26]. Studies also have shown that P2RX1 is closely related to the platelet activation pathway [27]. Phosphofructokinase platelet-type (PFKP) is a rate-limiting enzyme involved in glycolysis and is closely related to the progression of non-small cell lung cancer [28]. Excised nucleoside triphosphate diphosphate hydrolase (ENTPD) is involved in inflammation and platelet aggregation [29]. Studies have shown a high correlation between TIMP1 hemoglobin levels and *in vitro* platelet reactivity [30].

Correlation analysis shows that the high-risk group is positively correlated with tumor mutation load and PD-L1.

However, certain limitations of this study should also be noted. First, this study is only a retrospective analysis, and a prospective multicenter study should also be conducted. Second, we deleted the gene TREML1 because the validation group did not have it. However, in the forest plot, TREML1 has very strong statistical significance. And some studies have shown that TREML1 is a prognostic gene of prostate cancer. However, there is no research on TREML1-associated lung adenocarcinoma. Therefore, it is necessary to further experiment with the role of TREML1 in the tumor in future research.

## CONCLUSIONS

In conclusion, we have constructed and confirmed a prognostic risk scoring system for lung adenocarcinoma platelet receptor-related genes consisting of eight genes. And we provided a free nomogram prediction web tool for clinicians (https://wsxzaq.shinyapps.io/wsxzaq_nomogram/). According to the results of functional annotation, the high-risk group participates in the cell cycle, and the pathways related to platelet-derived growth factor and glycolysis are abundant. This may help to explain the molecular mechanism of lung adenocarcinoma occurrence and development. In addition, our data provide new and promising evidence for predicting lung adenocarcinoma biomarkers and targeted therapy.

## MATERIALS AND METHODS

### Clinical samples and data collection

RNA sequencing and clinical data were available from The Cancer Genome Atlas (TCGA: https://portal.gdc.cancer.gov/) and the Gene Expression Omnibus (GEO: https://www.ncbi.nlm.nih.gov/geo/) data portals. We adopted three datasets (GSE13213, GSE72094 and GSE68465) from the GEO database. We also populated a list of platelet-associated receptor genes from the GO Consortium database (http://geneontology.org/). The mutation spectrum and copy number variation (CNV) data for the entire TCGA dataset study population were also downloaded. Samples with less than 30 days of survival and samples with 0 expression values higher than 10% were removed.

### Analysis of differentially expressed genes

We performed differential gene analysis on all transcription data, setting the false discovery rate (FDR) to < 0.05, and |fold change| > 1.5 as the cutoff value [31]. The differentially expressed genes were then extracted from all the differentially expressed genes. Functional enrichment analysis was performed through the GO and KEGG pathways.

### Survival analysis

Survival related genes were selected by univariate and multivariate COX analysis, which was performed with R’s survival software package.

### Building a prognostic index based on platelet-related genes

We performed a multivariate analysis of core platelet-related genes to construct a platelet risk score (PRS). This was based on the expression data multiplied by the Cox regression coefficient. Patients were divided into high-risk and low-risk groups based on the median. The prognostic value of PRS was evaluated in patients with different subtypes. The TIMER database was used to analyze the abundance of tumor-infiltrating immune cells. The association was then calculated between PRS and LUAD immune cell infiltration.

### Gene set enrichment analysis

Gene set enrichment analysis (GSEA) is a computational method to identify over-represented biological processes and pathways.

### Mutation and copy number variation analysis

The genes were analyzed with different mutations in different risk score subtypes (high and low risks). The mutation spectrum of the entire study population from the TCGA dataset was determined as previously described. Copy number variation (CNV) data were downloaded from GDAC Firehose and divided into datasets according to the risk score expression.

### Gene expression and miRNA integration

To study microRNAs’ (miRNAs’) potential gene regulation, we focused on the abnormally expressed miRNAs (adjusted *p*-value < 0.05, log2 | multiple changes | > 1) and the RNAseq genes with statistically significant differences selected from high and low-risk patients. The correlation between miRNA and regulatory genes was obtained through TargetScan analysis (http://www.targetscan.org/vert_72/).

### Statistical analysis

The AUC for the survival ROC curve was calculated by the survivalROC R software package to verify the prognostic signature’s performance. Differences between clinical parameters were tested using independent *t*-tests. A *p*-value of less than 0.05 was considered statistically significant.

### Ethics approval and consent to participate

Our study did not require the approval of an ethics committee, as it was a secondary analysis of a public database.

### Data availability statement

The data that support the findings of this study are openly available in TCGA at https://cancergenome.nih.gov/, and GEO at https://www.ncbi.nlm.nih.gov/geo/, reference number: GSE13213, GSE68465 and GSE72094.

## Supplementary Material

Supplementary Figures
