# Supplementary material for: Predicting lung adenocarcinoma prognosis with a novel risk scoring based on platelet-related gene expression
Source: Aging (Albany NY). 2021 Feb 22;13(6):8706–19. doi: 10.18632/aging.202682 (PMC8034940; doi:10.18632/aging.202682)
Supplement: Supplementary Figures [file aging-13-202682-s001.pdf]

SUPPLEMENTARY FIGURES

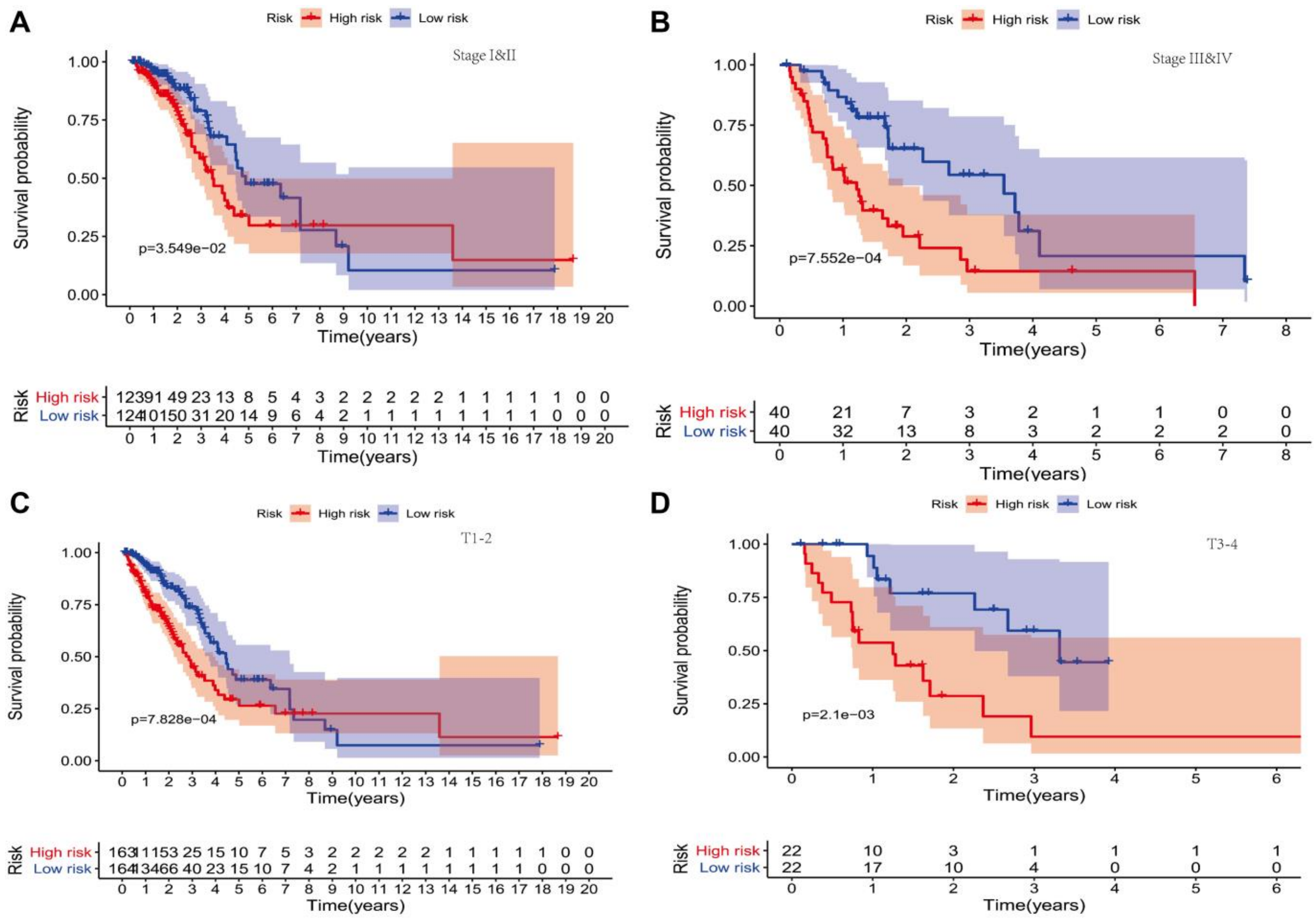

Supplementary Figure 1. Kaplan-Meier survival analysis according to the PRS in the TCGA cohort. (**A, B**) TNM stage. (**C, D**) T stage.

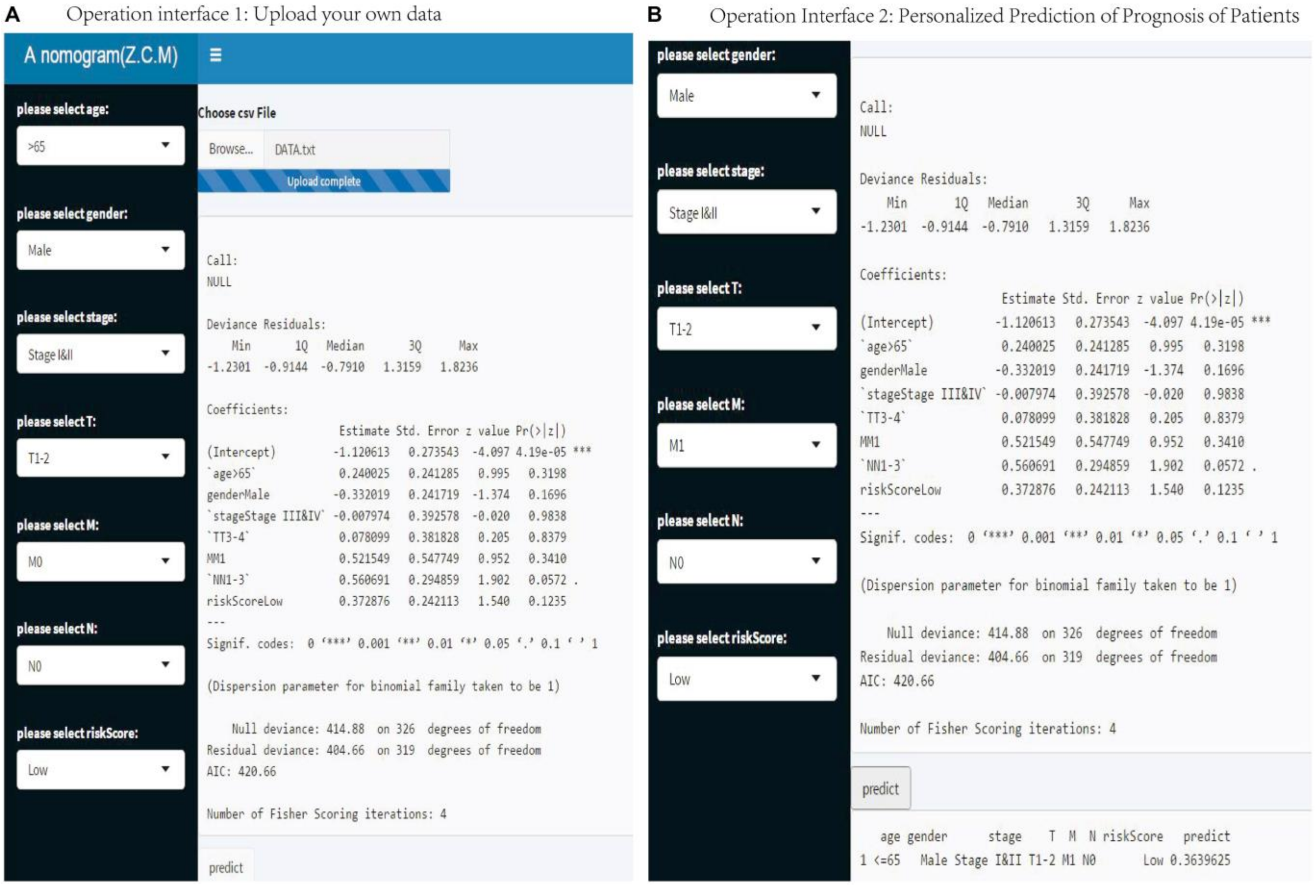

Supplementary Figure 2. Operating page of nomogram online web page prediction tool based on risk score and clinical characteristics. (**A**) Upload data operation page. (**B**) Personalized Selection of Patient Information Operation Interface.

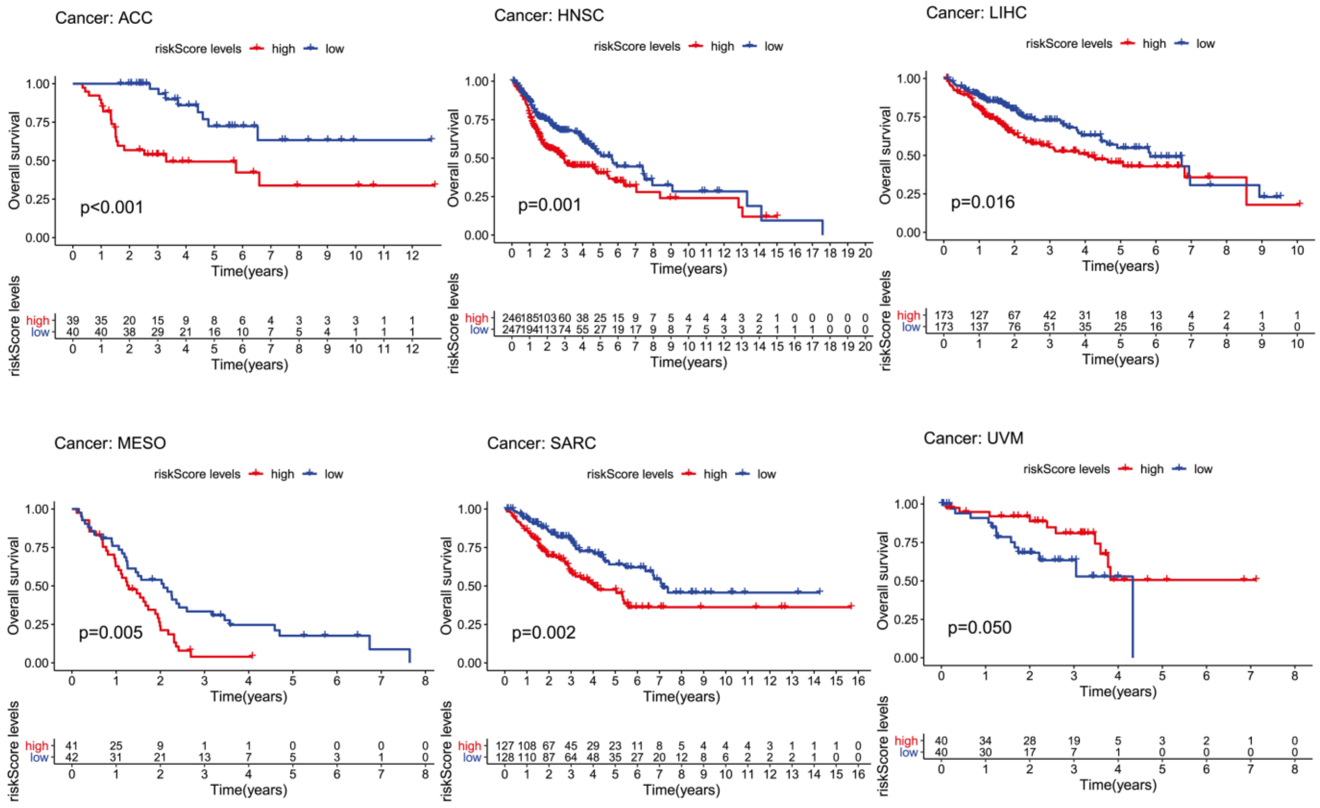

Supplementary Figure 3. Correlation between PRS and other 6 cancers.
